# Acute Physiological Responses to Different Load Components During Interval Training

**DOI:** 10.3390/sports14090405

**Published:** 2026-09-15

**Authors:** Daria Segev, Einat Kodesh, Ayelet Dunsky, Alon Eliakim, Yoav Meckel

**Affiliations:** 1 Department of Physical Therapy, Faculty of Social Welfare and Health Science, University of Haifa, Haifa 3498838, Israel; dariamsegev@gmail.com; 2 School of Human Movement and Sport Sciences, The Levinsky-Wingate Academic College, Netanya 42920200, Israel; ayelet@l-w.ac.il (A.D.); aloneliakim@gmail.com (A.E.); meckel@l-w.ac.il (Y.M.)

**Keywords:** anerobic training, training adjustment, exercise intensity, blood lactate

## Abstract

Interval training is a popular method for enhancing athletic performance, adjustable through speed, distance, and rest. The acute physiological and subjective responses to isolated manipulation of these components remain unclear. This study examined acute physiological and perceptual responses to systematic manipulation of speed, distance, or rest during interval training. Twenty-two young male physical-education students (average age 25.2 ± 3.5) with high baseline physical activity but no prior structured interval-training experience completed three randomized interval protocols. In all protocols, the first interval was identical (92% vVO_2_max, 250 m, 180 s rest). In the remaining five intervals, either speed, distance, or recovery duration was progressively changed in 8% steps (speed, distance, and rest protocols, respectively), while the other two components were kept constant. Blood lactate (La), oxygen consumption (VO_2_), ventilation (VE), respiratory exchange ratio (RER), heart rate (HR), and rating of perceived exertion (RPE) were measured throughout. Overall, the speed protocol elicited the highest average peak values across all measured parameters (*p* < 0.001). However, the percentage change in VO_2_ from Interval 1 to Interval 6 did not differ significantly between the speed and distance protocols. Between-protocol differences varied across outcomes: the speed protocol elicited greater changes in Lactate from Interval 2 onward and in VE and HR from Interval 3 onward (*p* < 0.05). Rate of perceived exertion was highest during the speed protocol (*p* < 0.001) but did not differ significantly between distance and recovery protocols. Applying the same relative 8% progression across interval components elicited distinct physiological responses. Progressive increases in running speed produced greater acute peak cardiorespiratory and metabolic responses than progressions in distance or reductions in recovery duration.

## 1. Introduction

Interval training is a widely used method for enhancing the physical fitness of professional and recreational-level athletes [1,2,3,4]. In this training method, exercise is performed at a high intensity level, in relatively short bouts of activity separated by recovery periods [2,3,5,6]. The key advantage of interval training is that it enables individuals to sustain intense exercise for a longer cumulative duration, thereby effectively stimulating and enhancing both aerobic and anerobic metabolic systems [4,5,6].

Interval training load is typically determined by several components, including interval speed, interval distance, or rest time between intervals [5]. Manipulating these load components may alter the overall training load and, consequently, elicit different physiological responses and training effects. Therefore, athletes and coaches may use different interval training strategies to generate the desired training load. Previous studies have explored various strategies to increase load during interval training [2,4,5,6,7,8,9,10]. One strategy is to gradually increase running speed or power output throughout the intervals. Other methods include increasing interval distance [11] or shortening recovery time to induce a greater training effect [3,5]. Interval-training protocols have varied widely across studies. For example, protocols such as sets of 30 s at all-out intensity separated by 4–4.5 min resting recovery periods are commonly used [1,12,13,14], while other training protocols use long and short intervals. High-intensity interval protocols vary in their structure: some prescribe work intervals at vVO_2_max using durations derived from a proportion of individual time-to-exhaustion with a 1:1 work-to-recovery ratio [15], while others utilize fixed-duration bouts ranging from 2 to 4 min [2]. Short intervals last 10–30 s at “all-out” intensities, with a passive or active rest for 2–4 min [3,14,16,17]. In addition, distance-based sprint protocols utilizing varying interval configurations (increasing vs. decreasing distances) have been reported. For instance, an acute protocol evaluated sprint runs over distances of 100 to 400 m at 80% of maximal speed, with rest periods between bouts ranging from 2 to 4 min (totaling 9 min of rest) [18]. In contrast, a 6-week training program employed sprint distances from 100 to 500 m at 75% of maximal speed, with rest periods scaling between 3 and 9 min [11]. However, the acute physiological and perceptual responses to progressively manipulating individual load components within a single protocol remain insufficiently characterized. To evaluate training intensity, different variables can be monitored throughout the activity. In a non-laboratory setting, measurements of heart rate (HR) [19,20] or rate of perceived exertion (RPE) [21] are commonly used as practical and accessible indicators of exercise intensity. For more precise intensity assessments, blood lactate concentration may also be measured [22,23]. In a laboratory setting, additional physiological indicators, including oxygen consumption (VO_2_), ventilation (VE) [20,23], and respiratory exchange ratio (RER) [24], may be monitored to quantify internal physiological load. Although strategies to increase interval training load (i.e., speed, distance, and rest) have been examined separately and used in various studies [2,11], the specific contribution of the present study is the direct within-participant comparison of three progressive interval protocols. In each protocol, speed, distance, or recovery duration was manipulated separately using the same percentage progression, while the other two components remained constant, allowing the resulting acute physiological and perceptual responses to be quantified and compared. Therefore, the present study aimed to compare the acute physiological and perceptual responses elicited by these three interval-progression strategies.

## 2. Materials and Methods

### 2.1. Participants

The sample size was determined a priori using G*Power (version 3.1.9.7), with V̇O_2_ selected as the primary physiological outcome based on previous evidence demonstrating its sensitivity to HIIT [4]. Because directly comparable acute crossover data were not available, a moderate effect size (f = 0.25) was assumed, with α = 0.05, 80% statistical power (β = 0.20), one group, three repeated measurements (Nmeasurements = 3), an assumed correlation among repeated measures of r = 0.50, and a nonsphericity correction of ε = 1.0.

A minimum of 20 participants was required. To account for potential attrition and ensure an adequate final sample (anticipated ~25% dropout rate across the multi-session trial), 27 participants were recruited. Of these, 22 completed all experimental protocols. Five participants withdrew (18.5% attrition): three withdrew voluntarily after the preliminary testing because of scheduling constraints, and two withdrew before completing the final experimental protocol for personal reasons. We excluded partial data from these participants from the analysis.

All participants were male physical-education students aged 20 to 36. The participants were routinely active in practical classes (e.g., ball games, track and field, gymnastics) as part of their academic program. Additionally, they averaged 8 h of physical activity per week, mostly playing ball games and engaging in aerobic training; however, none had previously participated in a structured interval training program. Inclusion criteria included physician approval to engage in physical activity and a 200 m maximal run time of <33 s. Baseline exclusion criteria included cardiac, pulmonary, or inflammatory diseases; other medical contraindications to high-intensity physical activity. For the final statistical analysis, data from 22 participants were included. Participation was entirely voluntary and independent of academic requirements. None of the participants were evaluated, graded, or directly supervised by the authors, and all participants were informed that declining participation or withdrawing from the study would have no academic consequences. The study was approved by the Ethics Committee of Meir Medical Center (approval no. 0243-21-MMC; 20 October 2022).

### 2.2. Study Procedure (Figure 1)

Before data collection, all participants received a detailed verbal and written explanation of the study’s purpose and procedures and provided written informed consent to participate. To ensure a relatively homogeneous group with comparable running qualities, participants first completed a screening test that included a 200 m all-out run. The test was conducted on a flat asphalt track after a standardized 10 min warm-up, which included light jogging and dynamic joint mobility exercises. All screening runs were conducted between 7:00 and 9:00 a.m., with 200 m sprint performance times ranging from 25.7 to 32.4 s (29.8 ± 1.9 s). Although a range of sprint performance was observed, all interval training workloads were strictly individualized relative to each subject’s physiological profile (specifically vVO_2_max). Monitoring during exercise confirmed that all participants exhibited uniform physiological response trends under identical relative demands, thus justifying their inclusion.

During the first laboratory visit, a graded exercise test was performed to determine VO_2_max. This test was conducted at least two days after the 200 m screening to minimize fatigue-related interference. The VO_2_max test was performed using a progressive incremental treadmill run (Technogym, Excite Run 1000, Cesena, Italy). After a standardized warm-up, the test began at 9 km/h, at a constant 1% gradient, and increased by 1 km/h every minute until volitional exhaustion. VO_2_max was measured breath-by-breath and was defined as the highest 30 s average of oxygen consumption recorded during the test. Attainment of VO_2_max was verified when at least two of the following criteria were met: (1) a plateau in VO_2_ despite an increase in workload; (2) a respiratory exchange ratio (RER) higher than 1.15; and (3) peak heart rate equal to at least 90% of the age-predicted maximum [calculated as 208 − (0.7 × age)]. All 22 participants met at least two criteria for attainment of maximal effort during the incremental exercise test. Specifically, 21 participants (95.5%) achieved the age-predicted maximal heart rate criterion, 18 (81.8%) achieved an RER > 1.15, and 13 (59.1%) demonstrated a VO_2_ plateau.

The order of the three interval protocols was randomized using computer-generated block randomization. All interval protocols were performed on the same treadmill.

### 2.3. Interval Training Protocols

**Figure 1:**
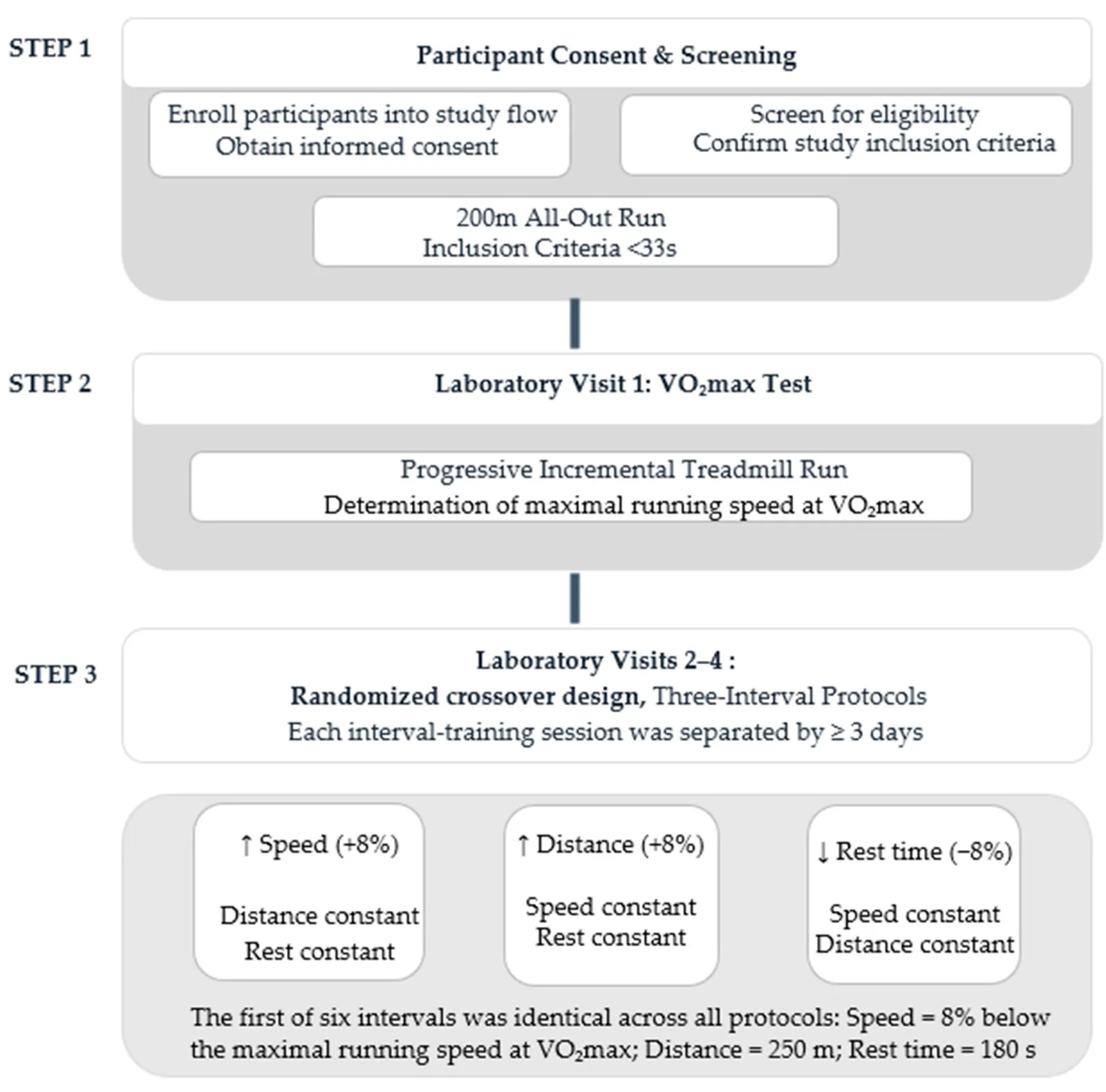
**Study design and experimental procedure**. The study consisted of three sequential steps: (1) participant screening and eligibility assessment, (2) a graded VO_2_max test, and (3) three randomized crossover interval-training protocols separated by ≥3 days.

The following laboratory visits consisted of three interval training sessions, each separated by at least three days to minimize residual fatigue (Figure 1). All three sessions were conducted at the same time of day for each participant under controlled environmental conditions (temperature 21–24 °C) on a treadmill (Technogym, Excite Run 1000, Cesena, Italy). During these visits, participants performed three distinct interval-training protocols designed to progressively manipulate a single load component in each protocol in a random order: (1) increasing running speed, (2) increasing running distance, or (3) decreasing rest duration between intervals. To minimize between-session variability, participants were instructed to maintain their usual diet, sleep schedule, and hydration and to avoid strenuous exercise for 24 h before each testing session. Habitual caffeine consumers were permitted to maintain their usual intake; however, all participants were instructed to refrain from food and caffeine for 2 h before testing, with water permitted. Participants verbally confirmed adherence upon arrival at the laboratory. No participant reported medication use during the study. The first interval (baseline) was identical across all three protocols, with running speed set at 92% of each participant’s vV̇O_2_max, a running distance of 250 m, and a recovery duration of 180 s.

The initial values and the 8% progression were determined through preliminary pilot testing. The purpose of the pilot testing was to identify a starting running intensity and a progressive increase that would meaningfully elevate exercise demand while allowing participants to complete all six intervals. An 8% increment met these criteria and was therefore selected.

Running speed was individualized using vVO_2_max as the physiological reference. In contrast, an established method for physiologically matching proportional changes in interval distance or recovery duration to changes in running speed was not available. Therefore, the same relative 8% progression was applied to each manipulated component using its own reference value: individual vV̇O_2_max for speed, the initial 250 m distance for distance, and the initial 180 s recovery duration for recovery. Only one load component (i.e., speed, distance, or rest time) was progressively modified across the subsequent intervals in each protocol, while the other two components remained constant within each protocol.

This approach was consistent with the exploratory aim of the study, which was to examine how acute physiological and perceptual responses differed when speed, distance, or recovery duration was progressively manipulated by the same relative amount, rather than to compare physiologically equivalent loads.

vV̇O_2_max was defined as the highest treadmill speed sustained for at least 30 s during the maximal graded exercise test; if the final stage was sustained for less than 30 s, the speed of the last completed stage was used. To ensure accurate implementation of the prescribed intervals, participants remained on the treadmill side rails until the belt reached the target running speed and then stepped onto the moving belt. Timing and distance recording began at that point. Recovery was standardized as active walking. During each recovery period, treadmill speed was immediately reduced to 4 km/h, and participants walked for the recovery duration. The treadmill fully accommodated all required velocities (up to 25 km/h). For safety during high-intensity intervals, all sessions were supervised by two research assistants: one operating treadmill controls and the second directly monitoring participant safety.

An example of the resulting progression across the six intervals for one participant with a vV̇O_2_max of 16 km/h is presented in Table 1. A more detailed presentation of the external load characteristics for the same illustrative participant across all three protocols is provided in Supplementary Table S1.

### 2.4. Physiological Variables

At the beginning of each session, participants wore a silicone face mask for gas collection and an HR chest-strap sensor (Polar Electro H7, BT Smart 4.0, Kempele, Finland). Then they completed a standardized five-minute warm-up on the treadmill at 8 km/h at a 1% incline. The treadmill incline was maintained at 1% throughout the graded exercise test and all experimental protocols to more closely approximate the energetic cost of outdoor running [25]. During each training protocol, HR was recorded continuously, and blood lactate concentration was measured via a finger prick using a lactate analyzer (Lactate Pro 2, LT-1730, Kyoto, Japan) and Lactate Pro 2 Test Strips, exactly one minute after the end of each interval. Participants briefly stepped onto the treadmill side rails for sampling and then resumed active recovery at 4 km/h. VO_2_ consumption, VE, and RER were measured breath-by-breath and analyzed using the Cortex Metalyzer 3B metabolic system and MetaSoft^®^ Studio software (Cortex Biophysik, Leipzig, Germany). Data were automatically averaged into fixed, non-overlapping 20 s time bins starting precisely from the onset of each active work bout (0–20 s, 20–40 s, etc.). Interval peak values were restricted strictly to the active work-bout duration to accurately isolate the maximal physiological strain from recovery dynamics. Daily calibration included ambient air, reference gas, and a 3 L syringe.

### 2.5. Subjective Variables

At the end of each interval, participants rated their perceived exertion using the Borg Category-Ratio 10 (CR10) scale, which ranges from 0 (“Nothing at all”) to 10 (“Extremely strong/Maximal”) [26].

### 2.6. Statistical Analysis

Statistical analyses were conducted using SPSS Statistics for Windows (Version 29.0). Continuous variables are presented as mean ± standard deviation. Normality was assessed for each Protocol × Interval cell distribution using the Kolmogorov–Smirnov test with Lilliefors correction and Monte Carlo significance (10,000 samples). Parametric normality assumptions held across the majority of cells for continuous physiological outcomes: for RPE and HR, deviations from normality occurred in 10/18 cells due to exertion ceiling effects.

To examine differences across conditions and over time, a Two-Way Repeated-Measures ANOVA was performed with two within-participant factors: Protocol (3 conditions) and Interval (6 time points), using interval peak values as inputs. For HR and RPE, which showed deviations from normality, a nonparametric sensitivity analysis using the Friedman test was also performed within each protocol to assess time-course changes. To additionally assess between-protocol differences nonparametrically, Friedman’s tests were conducted across the three protocols at each interval. Holm correction was applied across the six interval-specific Friedman tests, and Bonferroni-adjusted pairwise comparisons were used to identify specific between-protocol differences. In addition, One-Way Repeated-Measures ANOVAs were conducted to compare percentage changes from Interval 1 to Interval 6 (Δ%) across the three protocols. Percentage changes were calculated as $\left[\left(\mathrm{Interval}\;6-\mathrm{Interval}\;1\right)/\mathrm{Interval}\;1\;\times \;100\right]$ and then averaged across participants.

Sphericity was assessed using Mauchly’s test for each within-subject factor and their interaction; when sphericity was violated (*p* < 0.05), degrees of freedom were adjusted using the Greenhouse–Geisser correction. Testing sequence order was evaluated as a between-subject factor, confirming no significant protocol-order or carryover effects (*p* > 0.05). Effect sizes for the repeated-measures ANOVAs are reported as partial eta-squared (ηp^2^).

For pulmonary gas exchange VE, VO_2_, RER, HR, and RPE, a complete-case analysis was performed with no missing interval-level observations across all 22 participants and protocols. For blood lactate, 9 of 396 planned measurements (2.27%) were missing due to unsuccessful sampling in six participants. The repeated-measures ANOVA used listwise deletion and therefore included 16 participants with complete data. Lactate descriptive statistics were calculated for these same participants.

As a sensitivity analysis, a linear mixed model incorporated all 387 available lactate observations from 22 participants without imputation. Protocol, interval, and their interaction were included as fixed effects, with a compound-symmetry covariance structure to account for repeated measurements within participants. The model was fitted using restricted maximum likelihood estimation, with Satterthwaite approximation for denominator degrees of freedom.

## 3. Results

Of the 27 participants who started the study, 22 completed all intervals and training sessions. Three participants chose to retire voluntarily after the preliminary tests, and two withdrew from the study for personal reasons that prevented them from participating in the final training protocol. Participants’ characteristics are described in Table 2.

Mean values and standard deviations of all peak variables across intervals for each of the three protocols are presented in Table 3.

**Figure 2:**
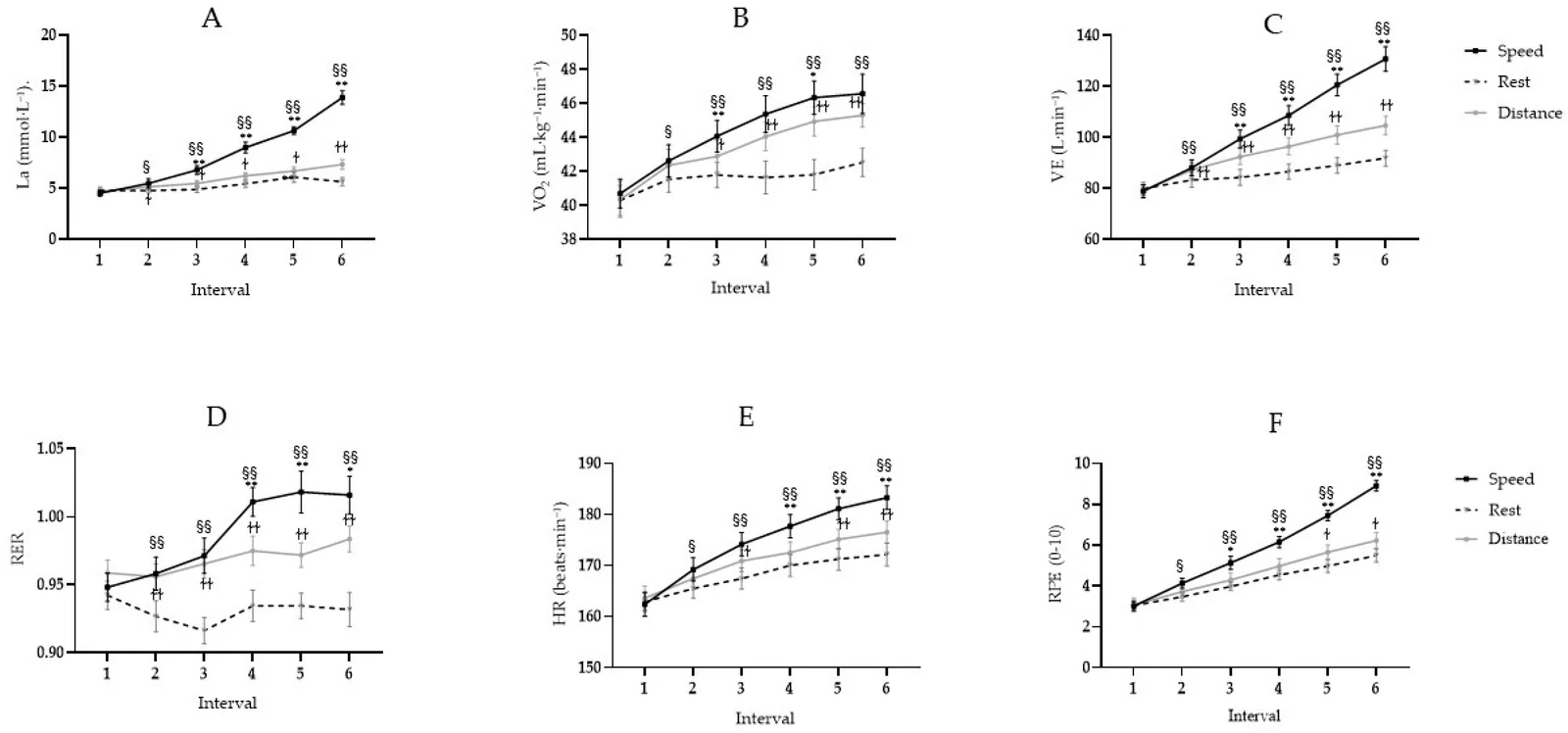
**Physiological and perceptual responses across the six consecutive intervals during the three interval-training protocols.** Changes in (**A**) blood lactate concentration (La), (**B**) oxygen consumption (VO*_2_*), (**C**) ventilation (V̇E), (**D**) respiratory exchange ratio (RER), (**E**) heart rate (HR), and (**F**) rating of perceived exertion (RPE) across the six consecutive intervals. Data are presented as mean peak values ± SE. The solid dark line represents the speed protocol, the solid light-gray line the distance protocol, and the dashed line the rest protocol. Symbols indicate significant pairwise comparisons following the two-way repeated-measures ANOVA: * *p* < 0.05 and ** *p* < 0.01 indicate speed vs. distance (positioned directly above the speed curve); § *p* < 0.05 and §§ *p* < 0.01 indicate speed vs. rest (positioned directly above the speed curve); † *p* < 0.05 and †† *p* < 0.01 indicate distance vs. rest (positioned directly adjacent to the distance curve, above or below depending on vertical spacing).

Overall, a significant protocol × interval interaction was observed for all physiological and subjective variables (all *p* < 0.001). All physiological and subjective variables differed significantly between protocols and intervals (all *p* values < 0.01), as shown in Figure 2A–F. The speed protocol consistently produced the highest responses. Post hoc pairwise comparisons confirmed that physiological and subjective values during Interval 1 did not differ significantly between protocols (*p* > 0.05), confirming equal baseline conditions.

As the protocols progressed, between-protocol differences emerged and varied across outcomes. The speed protocol generally elicited greater responses than the distance and recovery protocols, while the distance protocol generally produced higher physiological responses than the recovery protocol. Detailed interval-specific pairwise comparisons are presented in Figure 2. Non-parametric sensitivity analyses showed no significant between-protocol differences in HR or RPE at Intervals 1 and 2. Significant differences emerged from Interval 3 onward after Holm correction for both HR (χ^2^(2) = 14.48–25.40, adjusted *p* ≤ 0.002) and RPE (χ^2^(2) = 11.86–37.87, adjusted *p* ≤ 0.008). Bonferroni-adjusted pairwise comparisons showed higher HR in the speed than rest protocol at Interval 3 (*p* = 0.001) and Intervals 4–6 (all *p* < 0.001), and higher HR in the speed than distance protocol at Intervals 5 (*p* = 0.013) and 6 (*p* = 0.020). For RPE, the speed protocol was higher than the rest protocol at Interval 3 (*p* = 0.016) and Intervals 4–6 (all *p* < 0.001), and higher than the distance protocol at Intervals 3 (*p* = 0.025), 4 (*p* = 0.008), and 5–6 (both *p* < 0.001). No significant differences were detected between the distance and rest protocols for either HR or RPE.

**Figure 3:**
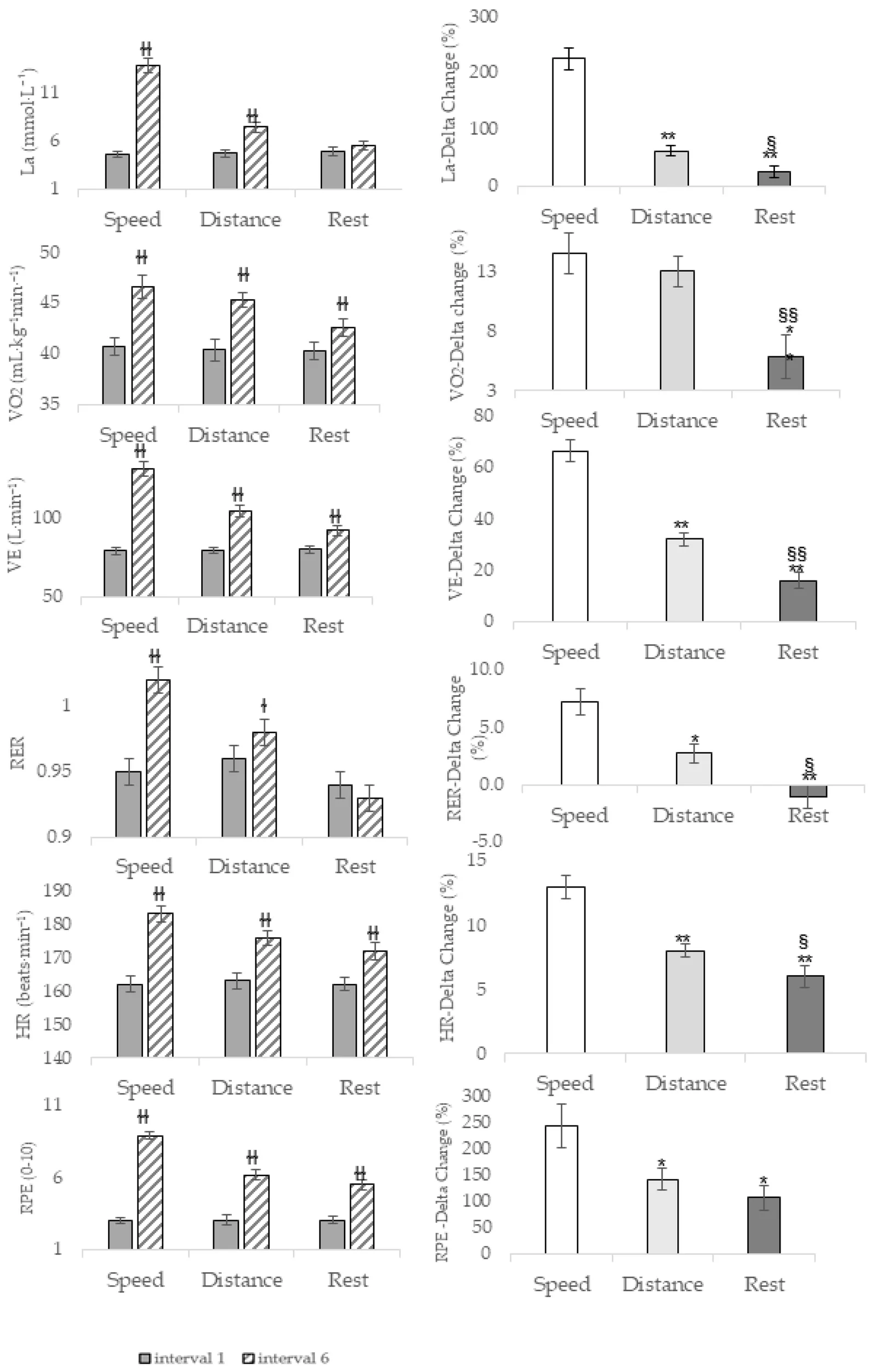
**Comparison of physiological and perceptual responses between Interval 1 and Interval 6 across the three protocols.** The left panels present the absolute values of blood lactate concentration (La), ventilation (V̇E), oxygen consumption (VO_2_), respiratory exchange ratio (RER), heart rate (HR), and rating of perceived exertion (RPE) measured at Interval 1 (light gray bars) and Interval 6 (hatched bars). The right panels present the corresponding percentage change from Interval 1 to Interval 6 for the speed (white bars), distance (light gray bars), and rest (dark gray bars) protocols. Data are presented as mean ± SE. * *p* < 0.05 and ** *p* < 0.01 versus the speed protocol; § *p* < 0.05 and §§ *p* < 0.01 versus the distance protocol; † *p* < 0.05 and †† *p* < 0.01 versus Interval 1.

Additionally, absolute values and percentage differences for all study variables between the first and last intervals are presented in Figure 3. Significant protocol effects were found for ΔLa, F(2,42) = 74.32, *p* < 0.001, ηp^2^ = 0.780; ΔVO_2_, F(2,42) = 10.66, *p* < 0.001, ηp^2^ = 0.337; ΔV̇E, F(1.54,32.38) = 97.76, *p* < 0.001, ηp^2^ = 0.823; ΔRER, F(2,42) = 18.07, *p* < 0.001, ηp^2^ = 0.462; ΔHR, F(2,42) = 38.99, *p* < 0.001, ηp^2^ = 0.650; and ΔRPE, F(1.37,28.77) = 8.92, *p* = 0.003, ηp^2^ = 0.298. Post hoc comparisons showed greater ΔLa in the speed than distance (*p* < 0.001) and rest (*p* = 0.002) protocols, and in the distance than rest protocol (*p* = 0.04). For ΔVO_2_, both the speed and distance protocols were greater than rest (both *p* < 0.001). For ΔV̇E, speed exceeded both distance and rest (both *p* < 0.001), and distance exceeded rest (*p* < 0.001). For ΔRER, speed exceeded distance (*p* < 0.001) and rest (*p* = 0.03), and distance exceeded rest (*p* = 0.02). For ΔHR, speed exceeded both distance and rest (both *p* < 0.001), and distance exceeded rest (*p* = 0.02). For ΔRPE, speed exceeded rest (*p* = 0.01) and distance (*p* = 0.03), with no significant difference between distance and rest.

## 4. Discussion

The present study extends previous research on interval-training configurations by directly comparing, within the same participants, the acute physiological and perceptual responses to three progressive protocols in which speed, distance, or recovery duration was manipulated separately using the same percentage progression. The main finding of the present study is that applying the same 8% relative manipulation to different interval-training components did not result in equivalent physiological responses. Under the specific progressive protocols examined, increasing running speed generally elicited greater physiological and perceptual responses than increasing distance or reducing recovery duration. Furthermore, manipulating interval distance led to significantly greater increases in all physiological variables than reducing rest duration between intervals, except for RPE. Thus, an equivalent percentage manipulation across interval-training components should not be interpreted as an equivalent physiological load.

Although load component manipulation in interval training has been widely discussed in the literature [5,6], most studies have focused on comparisons between distinct protocols rather than investigating within-session alterations. In addition, all three protocols resulted in elevated blood lactate concentrations, measured after the final interval: speed: 13.6 mmol/L; distance: 7.4 mmol/L; and rest: 5.5 mmol/L. In the speed protocol, blood lactate levels increased markedly to over 13.6 mmol/L, indicating substantially greater metabolic demand. Moreover, the speed protocol induced a pronounced elevation in VE and RER, with the most significant changes occurring from Interval 4. La, VE and RER provide valuable insight into the internal load imposed on the body during strenuous activity and serve as key markers for physiological strain and performance assessment [27,28]. As exercise intensity exceeds the anerobic threshold, reliance on anerobic glycolysis increases, resulting in greater metabolic acidosis and increased VE, which, in turn, contributes to a rise in RER values, usually exceeding 1.0 [29]. A consistent pattern was observed in the present study, with RER values reaching a mean of 0.99 ± 0.05 (Table 3) and reaching approximately 1.02 during the final interval (Figure 2D). The relatively modest RER response may partly reflect the short interval duration and the kinetics of pulmonary gas exchange [30]. The concurrent increases in V̇E and La further reflect the increasing anerobic contribution and physiological demands of the exercise [23].

When rest duration was reduced by 8% while maintaining a constant distance of 250 m and a speed of 8% below vVO_2_max, the lowest values of La, VE, and RER were observed. Moreover, no significant differences were observed between the first and last intervals for the La and RER variables, reflecting stable responses across intervals without progressive increases in these physiological variables. These findings align with previous evidence indicating that substantial lactate accumulation may occur during maximal or supramaximal interval exercise (≥vVO_2_max) when inter-interval recovery periods are short, such as 30 s [2,31]. In contrast, when exercise is performed at submaximal intensity, lactate accumulation remains limited even with reducing rest duration between intervals [2]. These findings suggest that an 8% progressive reduction in recovery duration was insufficient to produce a physiological response comparable to that elicited by the 8% progression in running speed. A larger reduction in recovery duration may therefore be required to achieve a comparable physiological stimulus; however, this requires direct investigation.

The VO_2_ responses showed a consistent change from the initial to the final interval (ΔVO_2_) across all three protocols. Despite this, the average peak VO_2_ values throughout the session were significantly higher in the speed protocol. However, this difference was not consistent across individual intervals, with significantly higher VO_2_ in the speed protocol compared with the distance protocol observed only during Intervals 3 and 5. Specifically, while the final interval of the distance protocol (350 m at constant speed) achieved a comparable VO_2_ to the speed protocol (250 m, with speeds ranging from 18.4 to 23.6 km/h across participants), the overall average peak VO_2_ response across the session remained significantly greater with increased speed. Thus, the present findings do not indicate a uniform superiority of speed for eliciting VO_2_ responses across all intervals. While direct quantitative comparison is limited because summary metrics differ (session-mean VO_2_ vs. our interval-peak averages), similar conceptual patterns exist in the literature. Specifically, intervals performed at the velocity associated with VO_2_max were reported to elicit higher session-mean oxygen uptake (91.4% of maximum) compared to longer intervals, such as 4 × 4 min (80.4%) and 4 × 1000 m (83%) [32]. Another important aspect, reported in the literature, is the kinetics of oxygen consumption, which describes the rate at which VO_2_ increases toward maximal levels at the onset of exercise or during transitions from rest to exertion, particularly in interval training. The primary time constant for this response typically ranges from 20 to 35 s, reflecting how quickly the body can adjust its oxygen consumption during these phases [5]. Differences in work-bout duration may therefore partly contribute to the VO_2_ response observed in the present study. Increasing distance progressively prolonged the exercise interval, providing more time for VO_2_ to rise, whereas increasing speed reduced the time required to complete the fixed 250 m distance. However, this potential explanation was not directly tested and should be interpreted cautiously. While not directly assessed in the present study, it may be hypothesized that individuals with slow kinetics may experience a delayed increase in oxygen consumption, which can hinder their ability to reach the necessary intensity for optimal performance. This slower response may be influenced by factors such as physical conditioning, muscle fiber composition, and metabolic efficiency [2,5]. As a result, incorporating speed into training can be particularly beneficial for those with slower kinetics, as it may allow them to reach the required intensity levels more quickly than using distance or rest alone. Finally, the acute testing conducted in the present study may have implications for interval training programs when determining whether to prioritize increases in interval’s distance or speed. Previous research has suggested that high-speed or maximal efforts may elicit distinct improvements in aerobic capacity compared with longer, more moderate intervals, depending on overall protocol parameters [7,16,32,33]. For example, repeated 30 s all-out cycling intervals induced significant increases in maximal mitochondrial respiration and key transcriptional regulators of mitochondrial biogenesis (e.g., PGC-1α), despite no observed changes in total mitochondrial content [16]. Similarly, our findings suggest that increasing load through speed, and thus creating shorter intervals, could lead to a similar or even higher acute oxygen-uptake response. In contrast, the findings of the present study suggest that increasing interval distance provides a more moderate approach to increasing training load. Although exercise duration is longer and running speed is lower, this strategy still elicits a significant increase in oxygen consumption while imposing a more moderate physiological stimulus than increasing running speed does. This response remains influenced by total work, recovery structure, and participant training status. Differences in interval duration and total work volume may therefore partially explain the observed ${\mathrm{VO}}_{2}$ responses in the present study.

In line with these findings, increasing running speed in the present study led to greater perceived effort than with other load components. In contrast, no significant differences in RPE were observed between the rest and distance protocols, despite clear differences in physiological markers, suggesting that extending running distance increased physiological strain without increasing perceived effort relative to reduced rest duration (Table 3). Given the physiological responses, the consistently higher RPE values observed during the speed protocol are consistent with the greater physiological demands imposed by this condition. This finding aligns with previous studies reporting higher RPE at greater running velocities, including vV̇O_2_max [32], and with interval training protocols associated with greater metabolic stress. However, blood lactate accumulation and RPE should not be interpreted as having a direct or linear relationship [34]. The lack of significant RPE differences between rest and distance protocols, despite clear distinctions in physiological markers (La, VO_2_, VE, RER, and HR), suggests RPE is not solely governed by physiological load. Unlike previous research evaluating perceptual strain across static or pyramid interval structures, the present findings demonstrate that this perceptual–physiological dissociation persists during acute, single-variable load progressions [11,35]. The present findings demonstrate that this perceptual–physiological dissociation also persists during acute, single-variable load progressions. A possible explanation lies in psychological factors and in perceived effort. A study that observed a descending-load interval protocol, while physiologically more demanding, yielded lower RPE than an ascending-load protocol, which was attributed to a sense of accomplishment [35]. Similarly, in our study, the progressive increase in difficulty (through increased distance or decreased rest) likely modulated RPE, supporting the notion that RPE is influenced by expectation, motivation, and perceived progress throughout the session [5,32,35].

Finally, these findings offer preliminary insights into how different interval-training components may be selected and progressed to elicit distinct acute physiological and perceptual responses. Under the present conditions, progression of running speed elicited greater overall acute demands than progression of distance or reduction in recovery duration. However, because post-session fatigue, recovery kinetics, subsequent performance, adherence, and chronic adaptations were not assessed, the implications of these acute differences for training prescription require further investigation in applied and longitudinal studies.

### Limitations

The present study employed a unique, structured interval training protocol designed to examine the acute physiological responses to manipulating incremental load components within a single session, although several limitations should be acknowledged. As a first step in testing this novel framework, a relatively homogeneous sample of recreationally active males was selected to minimize physiological variability. Accordingly, the findings may not generalize to females, highly trained athletes, or populations differing substantially in age, activity level, or health status. Although the same relative change of 8% progression was applied to speed, distance, and recovery duration, these manipulations were not physiologically matched. Speed progression was individualized relative to vV̇O_2_max, whereas distance and recovery duration were modified relative to their respective initial protocol values. Consequently, comparisons between protocols reflect responses to equivalent relative manipulations rather than equivalent physiological loads. Future studies should determine what proportional changes in each component are required to elicit comparable physiological demands. This study, focusing solely on acute physiological responses, necessitates longitudinal research to understand chronic adaptations and performance changes. While offering a novel, general comparison of load manipulation, its broad design highlights the need for further specific investigation. Future work should systematically compare speed, distance, and rest within specific aerobic and anerobic interval protocols and determine the component-specific magnitude of change required to produce comparable physiological loading to develop targeted prescription guidelines.

## 5. Conclusions

This acute study of within-session load progression during interval training demonstrates that in young active males, applying the same relative 8% manipulations of different interval-training components do not produce equivalent acute physiological responses. Increasing running speed generally produced greater acute peak cardiorespiratory and metabolic responses than increasing distance or reducing recovery duration, although this pattern was not consistent for VO_2_ across all intervals. The study quantifies the magnitude and pattern of these differences under the distinct running-speed, total-distance, and recovery demands resulting from the 8% progressions. These findings indicate that equivalent percentage changes across interval-training components should not be interpreted as equivalent physiological loads. Future studies are needed to determine the component-specific magnitude of change required to achieve comparable physiological loading and whether these acute differences translate into distinct long-term training adaptations.

## Figures and Tables

**Table 1:** Example of the progressive manipulation of training load across the three interval training protocols.

Protocol	Interval 1	Interval 2	Interval 3	Interval 4	Interval 5	Interval 6
Speed (km/h)	14.7 *	16	17.3	18.6	19.9	21.2
Distance (m)	250	270	290	310	330	350
Rest time (s)	180	166	152	138	124	110

Values illustrate one participant’s progression across the six intervals. In each protocol, only one load component was varied by 8% between consecutive intervals, while the other two components were held constant. The 8% progression was applied additively as a fixed absolute increment or decrement at each subsequent interval and was not compounded. * Baseline running speed was set at 8% below the participant’s vV̇O_2_max (16 km/h in this example). In the speed protocol, the 8% progression corresponded to 1.28 km/h, rounded to 1.3 km/h per interval, resulting in a final speed of 21.2 km/h (132.5% of vV̇O_2_max). In the distance protocol, running distance increased by 20 m per interval, reaching 350 m in Interval 6. In the recovery protocol, recovery duration decreased by 14 s per interval (14.4 s rounded to 14 s), reaching 110 s in Interval 6.

**Table 2:** Participant characteristics (*n* = 22).

Variables	Mean ± SD	Minimum	Maximum
Age (years)	25.2 ± 3.5	20	36
Weight (kg)	70.5 ± 7.6	58	83
Height (cm)	174.1 ± 6.8	158	189
200 m run time (s)	29.83 ± 1.9	25.7	32.4
VO_2_max (mL/kg/min)	52.7 ± 4.8	44.5	63.6
VEmax (L/min)	135.9 ± 20.4	90.9	163.6
HRmax (bpm)	187 ± 9.5	161	206
Baseline Speed (km/h)	14.9 ± 1.0	12.9	16.6
vVO_2_max (km/h)	16.1 ± 1.0	14	18
Interval 6 speed (km/h)	21.2 ± 1.4	18.4	23.6

Values are presented as mean ± SD. VO_2_max, maximal oxygen uptake; VEmax, maximal ventilation; HRmax, maximal heart rate; vVO_2_max, velocity at maximal oxygen consumption. Baseline speed was defined as 8% below the maximal running speed achieved during the graded maximal exercise test. Interval 6 speed represents the final prescribed running speed in the Speed protocol based on treadmill-rounded 8% increments.

**Table 3:** Comparison of average peak physiological and perceptual responses across the three interval training protocols.

				Repeated-Measures ANOVA			
Variable	Speed Protocol	Distance Protocol	Rest Protocol	Interaction	Interval	Protocol	Post Hoc Comparisons
**La** **(mmol/L)**	8.4 ± 1.67	5.9 ± 1.35	5.3 ± 1.34	F(_3.9,59.2_) = 31.97, *p* < 0.001, $\eta_{p}^{2}$ = 0.68	F(_2.3,34.5_) = 68.53, *p* < 0.001, $\eta_{p}^{2}$ = 0.82	F(_2,30_) = 126.28, *p* < 0.001, $\eta_{p}^{2}$ = 0.89	Speed > Distance > Rest
**VO_2_ (mL/kg/min)**	44.28 ± 4.36	43.32 ± 3.73	41.61 ± 3.94	F(_10,210_) = 7.15, *p* < 0.001, $\eta_{p}^{2}$ = 0.25	F(_5,105_) = 55.07, *p* < 0.001, $\eta_{p}^{2}$ = 0.72	F(_2,42_) = 37.89, *p* < 0.001, $\eta_{p}^{2}$ = 0.64	Speed > Distance > Rest
**VE** **(L/min)**	104.41 ± 16.31	93.51 ± 14.11	85.83 ± 13.1	F(_4.1,84.9_) = 66.11, *p* < 0.001, $\eta_{p}^{2}$ = 0.76	F(_1.8,37.9_) = 149.40, *p* < 0.001, $\eta_{p}^{2}$ = 0.88	F(_1.5,31.3_) = 112.20, *p* < 0.001, $\eta_{p}^{2}$ = 0.84	Speed > Distance > Rest
**RER**	0.99 ± 0.05	0.97 ± 0.04	0.93 ± 0.05	F(_10,210_) = 8.72, *p* < 0.001, $\eta_{p}^{2}$ = 0.29	F(_5,105_) = 15.35, *p* < 0.001, $\eta_{p}^{2}$ = 0.42	F(_2,42_) = 25.40, *p* < 0.001, $\eta_{p}^{2}$ = 0.55	Speed > Distance > Rest
* HR **(beat/min)**	175 ± 10.39	171 ± 9.62	168 ± 9.55	F(_4.2,87.8_)=25.4, *p* < 0.001, $\eta_{p}^{2}$ = 0.55	F(_1.4,30_) = 163.43, *p* < 0.001, $\eta_{p}^{2}$ = 0.89	F(_2,42_) = 10.71, *p* < 0.001, $\eta_{p}^{2}$ = 0.34	Speed > Distance > Rest
* RPE **(0–10)**	5.8 ± 1.06	4.7 ± 1.55	4.3±1.1	F(_4.9,103_) = 36.62, *p* < 0.001, $\eta_{p}^{2}$ = 0.64	F(_1.7,36.3_) = 193.93, *p* < 0.001, $\eta_{p}^{2}$ = 0.9	F(_2,42_) = 23.07, *p* < 0.001, $\eta_{p}^{2}$ = 0.52	Speed > Distance = Rest

Data are presented as mean ± SD. Statistical results are reported as F values, corresponding *p* values, and partial eta squared (ηp^2^) from repeated-measures ANOVA. Post hoc comparisons indicate statistically significant differences between protocols. La, blood lactate; VO_2_, oxygen consumption; V̇E, ventilation; RER, respiratory exchange ratio; HR, heart rate; RPE, rating of perceived exertion. Degrees of freedom (Greenhouse–Geisser adjusted where sphericity was violated). Peak values for oxygen uptake, Minute Ventilation, and respiratory exchange ratio represent the mean ± standard deviation of the highest 20 s average recorded during the active interval duration via breath-by-breath analysis. * Friedman analyses confirmed significant differences across intervals for HR: χ^2^(5) = 86.61–107.08, all *p* < 0.001 and RPE (χ^2^(5) = 89.64–107.58, all *p* < 0.001) within all three protocols; post hoc comparisons identified the specific interval differences. Additional between-protocol Friedman’s analyses showed significant differences from Interval 3 onward for both HR and RPE after Holm correction.

## Data Availability

The data supporting the findings of this study are available from the corresponding author upon reasonable request.

## Supplementary Materials

The following supporting information can be downloaded at: https://www.mdpi.com/article/10.3390/sports14090405/s1, Table S1: External-load characteristics of the illustrative protocol.

## Abbreviations

The following abbreviations are used in this manuscript:

ANOVA	Analysis of Variance
HIIT	High-Intensity Interval Training
HR	Heart Rate
HRmax	Maximal Heart Rate
La	Blood Lactate
RER	Respiratory Exchange Ratio
RPE	Rating of Perceived Exertion
VE,	Minute Ventilation
VO_2_	Oxygen Consumption
VO_2_max	Maximal Oxygen Consumption
vVO_2_max	Velocity at Maximal Oxygen Consumption
