# Supplementary material for: Acute Physiological Responses to Different Load Components During Interval Training
Source: Sports (Basel). 2026 Sep 15;14(9):405. doi: 10.3390/sports14090405 (PMC13611252; doi:10.3390/sports14090405)
Supplement: Supplementary file 1 [file sports-14-00405-s001.zip › sports-4463105-supplementary.pdf]

Supplementary Table S1. External-load characteristics of the illustrative protocol

An example of the resulting progression across the six intervals for one participant with a vVO_2max of 16 km/h

Speed protocol

Interval	Speed (km/h)	Distance (m)	Calculated work-bout duration (s)	Recovery after interval (s)	Cumulative distance (m)	Cumulative work time (s)	Work: recovery
1	14.7	250	61.2	180	250	61.2	0.34
2	16.0	250	56.3	180	500	117.5	0.31
3	17.3	250	52.0	180	750	169.5	0.29
4	18.6	250	48.4	180	1,000	217.9	0.27
5	19.9	250	45.2	180	1,250	263.1	0.25
6	21.2	250	42.5	—	1,500	305.6	—

Total running distance: 1,500 m; **Total calculated work time:** 305.6 s; **Total inter-interval recovery:** 900 s
Total calculated protocol time: 1,205.6 s; **Overall work: recovery ratio:** 0.34.

Distance protocol

Interval	Speed (km/h)	Distance (m)	Calculated work-bout duration (s)	Recovery after interval (s)	Cumulative distance (m)	Cumulative work time (s)	Work: recovery
1	14.7	250	61.2	180	250	61.2	0.34
2	14.7	270	66.1	180	520	127.3	0.37
3	14.7	290	71.0	180	810	198.4	0.39
4	14.7	310	75.9	180	1,120	274.3	0.42
5	14.7	330	80.8	180	1,450	355.1	0.45
6	14.7	350	85.7	—	1,800	440.8	—

Total running distance: 1,800 m; **Total calculated work time:** 440.8 s; **Total inter-interval recovery:** 900 s
Total calculated protocol time: 1,340.8 s; **Overall work: recovery ratio:** 0.49.

Recovery protocol

Interval	Speed (km/h)	Distance (m)	Calculated work-bout duration (s)	Recovery after interval (s)	Cumulative distance (m)	Cumulative work time (s)	Work: recovery
1	14.7	250	61.2	180	250	61.2	0.34
2	14.7	250	61.2	166	500	122.4	0.37
3	14.7	250	61.2	152	750	183.7	0.40
4	14.7	250	61.2	138	1,000	244.9	0.44
5	14.7	250	61.2	124	1,250	306.1	0.49
6	14.7	250	61.2	—	1,500	367.3	—

Total running distance: 1,500 m; **Total calculated work time:** 367.3 s; **Total inter-interval recovery:** 760 s
Total calculated protocol time: 1,127.3 s; **Overall work: recovery ratio:** 0.48
